# Normative data for two challenging tests of face matching under ecological conditions

**DOI:** 10.1186/s41235-019-0205-0

**Published:** 2020-02-19

**Authors:** Lisa Stacchi, Eva Huguenin-Elie, Roberto Caldara, Meike Ramon

**Affiliations:** 1 Eye and Brain Mapping Laboratory, Department of Psychology, University of Fribourg, Fribourg, Switzerland; 2 Applied Face Cognition Lab, Department of Psychology, University of Fribourg, Fribourg, Switzerland

**Keywords:** Superior face processing ability, Face perception, Face matching, Face discrimination, Face recognition, Natural image variations

## Abstract

**Background:**

Unfamiliar face processing is an ability that varies considerably between individuals. Numerous studies have aimed to identify its underlying determinants using controlled experimental procedures. While such tests can isolate variables that influence face processing, they usually involve somewhat unrealistic situations and optimized face images as stimulus material. As a consequence, the extent to which the performance observed under laboratory settings is informative for predicting real-life proficiency remains unclear.

**Results:**

We present normative data for two ecologically valid but underused tests of face matching: the Yearbook Test (YBT) and the Facial Identity Card Sorting Test (FICST). The YBT (*n* = 252) measures identity matching across substantial age-related changes in facial appearance, while the FICST (*n* = 218) assesses the ability to process unfamiliar facial identity despite superficial image variations. To determine the predictive value of both tests, a subsample of our cohort (*n* = 181) also completed a commonly used test of face recognition and two tests of face perception (the long form of the Cambridge Face Memory Test (CFMT+), the Expertise in Facial Comparison Test (EFCT) and the Person Identification Challenge Test (PICT)).

**Conclusions:**

Focusing on the top performers identified independently per test, we made two important observations: 1) YBT and FICST performance can predict CFMT+ scores and vice versa; and 2) EFCT and PICT scores neither reliably predict superior performance in ecologically meaningful and challenging tests of face matching, nor in the most commonly used test of face recognition. These findings emphasize the necessity for using challenging and ecologically relevant, and thus highly sensitive, tasks of unfamiliar face processing to identify high-performing individuals in the normal population.

## Significance Statement

How do humans process unfamiliar faces, and how can we reliably identify individuals that are most proficient at it? Motivated by its relevance in applied contexts, recent empirical work has sought to answer these questions. Controlled laboratory tests have been developed to understand the contribution of different variables and differences between observers. However, face processing tests involving face stimuli derived from ideal and highly controlled manipulations may not accurately represent real life. This crucial consideration is often overlooked when laboratory tests are used to predict real-life proficiency. The present study followed the rationale that traditionally used controlled tests should be paired with more realistic and ecologically meaningful ones, in terms of the images used and tasks performed. Testing large and heterogeneous samples, we standardized two less frequently used tests of facial identity matching: the Yearbook Test (YBT) and the Facial Identity Card Sorting Test (FICST). These procedurally simple tests mimic real-life challenges in face perception as they assess unfamiliar facial identity matching across superficial image changes, or substantial age-related changes in facial appearance. Beyond providing normative data, we describe how performance measured by these tests relates to that observed on more commonly used tests of face recognition (the long form of the Cambridge Face Memory Test (CFMT+)) and perception (the Expertise in Facial Comparison Test (EFCT) and the Person Identification Challenge test (PICT)). Our findings suggest that: 1) the YBT and FICST are easy to use and are preferable alternatives to pairwise face-matching tasks which are more prone to speed–accuracy trade-offs and/or ceiling effects; and 2) challenging and ecologically meaningful tests should complement highly controlled measures when aiming to identify individuals with superior face processing abilities for real-life purposes.

## Background

In our everyday lives, we effortlessly process constantly changing facial information. The commonly accepted notion that it is performed in a highly proficient manner underlies a vast body of research and, for the most part, it coincides with our own personal experiences. However, an increasing number of studies is challenging the idea that face processing is generally highly proficient. Extremely high performance levels are observed in particular for the processing of faces of individuals encountered repeatedly in everyday life—those that are personally familiar to us (for a review see Ramon & Gobbini, 2018). For unfamiliar faces, on the other hand, our ability to process facial identity is comparably more prone to error (e.g., Bruce, Henderson, Newman, & Burton, 2001; Burton, Wilson, Cowan, & Bruce, 1999; Hancock, Bruce, & Burton, 2000; Jenkins & Burton, 2011; Jenkins, White, Van Montfort, & Burton, 2011; Megreya & Burton, 2006; Ramon & Gobbini, 2018; Ramon & Van Belle, 2016; Young & Burton, 2017) and varies considerably between individuals (e.g., Bate & Dudfield, 2019; Bate, Portch, Mestry, & Bennetts, 2019; Bruce, Bindemann, & Lander, 2018; Fysh, 2018; White, Kemp, Jenkins, Matheson, & Burton, 2014).

Decades of research has focused on studying normal face processing skills, as well as individuals exhibiting deficient face processing abilities (i.e., developmental prosopagnosia; for a recent review see Geskin & Behrmann, 2018). However, more recently increasing interest has been directed toward individuals with remarkable face processing abilities—so-called super-recognizers (SRs; e.g., Ramon, Bobak, & White, 2019a, 2019b). Understanding superior face processing skills is important from both a fundamental scientific as well as an applied perspective. Theoretically, this work has led to face processing being considered as a spectrum rather than supporting a dichotomous distinction between normal and dysfunctional abilities (Russell, Duchaine, & Nakayama, 2009). From a practical perspective, investigation of the abilities of SRs can provide valuable information for the optimization of automatic face processing or deployment of personnel in security-critical settings (Ramon et al., 2019a, 2019b), such as criminal investigation (Ramon, 2018a).

However, a fundamental challenge continues to exist: How do we reliably identify high performing individuals within the general population? Addressing this issue requires consideration of aspects that have been expressed by scientists and practitioners alike (cf. Bate et al., 2019; Moreton, Pike, & Havard, 2019; Ramon et al., 2019a, 2019b; Robertson & Bindemann, 2019). Two major factors are: 1) assessment of different subprocesses or processing levels in face cognition (Ramon et al., 2019a; Ramon & Gobbini, 2018); and 2) the degree to which this process-dependent behavior observed experimentally translates into extremely varied and constantly changing applied settings (cf. e.g., Bate et al., 2019; Ramon, 2018a). These two factors are discussed below.

### Assessment of face perception versus recognition: procedural considerations

Concerning the first factor, distinct subprocesses involved in face cognition can be investigated through specific tasks that are (ideally) designed to capture them in a reliable manner. In the context of unfamiliar face processing, the most commonly assessed processes include face perception and face recognition. These are separate processes that require careful terminological distinction (cf. Ramon, 2018a; Ramon et al., 2019a; Ramon & Gobbini, 2018) and should not be used interchangeably or considered analogous to other processes (such as face identification; see Noyes, Hill, & O’Toole, 2018; Phillips et al., 2018).

Tests of *face perception* can involve simultaneous matching (or discrimination) among image pairs (e.g., Fysh, 2018; Ramon & Rossion, 2010), triplets (e.g., Barton, Press, Keenan, & O’Connor, 2002; Busigny, Joubert, Felician, Ceccaldi, & Rossion, 2010), 1-to-n matching scenarios (e.g., Bruce et al., 1999; Pachai, Sekuler, Bennett, Schyns, & Ramon, 2017; Rezlescu, Pitcher, & Duchaine, 2012), or can comprise delayed matching (e.g., Ramon, Busigny, & Rossion, 2010; Ramon & Van Belle, 2016). In the context of such laboratory-based face perception tests, consideration of both accuracy and reaction times (RTs) is necessary to accurately characterize individual face processing abilities. This is especially important for tests that 1) involve behavioral decisions recorded for individual trials with long or unlimited duration, and/or 2) are insufficiently calibrated to clear two standard deviations from the control mean (e.g., the Glasgow Face Matching Test (GFMT); Bate et al., 2018; Bobak et al, 2016a; Burton, White, & McNeill, 2010; Fysh, 2018; Fysh & Bindemann, 2018; Robertson et al., 2016; White, Phillips, Hahn, Hill, & O’Toole, 2015). To illustrate, time-consuming piecemeal matching strategies may enable normal performance levels in terms of accuracy even in highly impaired clinical populations, such individuals suffering from prosopagnosia (cf. Marotta, McKeeff, & Behrmann, 2002; White, Rivolta, Mike Burton, Al-Janabi, & Palermo, 2017).

Tests of *face recognition* involve intentional learning of previously unfamiliar identities, which are later recognized as “old” among novel ones (e.g., Bate et al., 2018; Bobak et al., 2016b; Russell et al., 2009). In the context of such face recognition tests, where encoding duration is classically predetermined, longer decision times cannot compensate for an inefficiently encoded face stimulus. Given the decreased likelihood of speed–accuracy trade-offs in face recognition tests, RTs are oftentimes not considered (e.g., Blais, Jack, Scheepers, Fiset, & Caldara, 2008).

To summarize, while face perception is usually assessed via matching of faces presented simultaneously, face recognition involves distinguishing experimentally learned identities from entirely novel ones. Importantly, observers can differ in their unique abilities exhibited across these distinguishable subprocesses (Fysh, 2018). Consequently, a comprehensive empirical understanding of the face processing abilities of individuals requires assessment across various levels of processing. In applied settings, on the other hand, this may not be required as, depending on the area of intended deployment, assessment of an ability confined to a specific type of task might be entirely sufficient (Bate et al., 2019; Moreton et al., 2019; Robertson & Bindemann, 2019).

### Relationship between performance in the laboratory and the real world

The second factor concerns the relationship between experimentally observed process-dependent behavior and skills that are relevant in extremely varied and constantly changing real-life settings (Moreton et al., 2019; Ramon et al., 2019a, 2019b). For the majority, empirical studies aim to characterize different aspects of face processing in a highly controlled manner. For instance, psychophysical studies of identity matching have been conducted to better understand the specific contribution of certain controllable factors—for example, low spatial frequency information or orientation tuning (Pachai, Sekuler, & Bennett, 2013; Pachai et al., 2017; Watier & Collin, 2009; see also Papinutto, Lao, Ramon, Caldara, & Miellet, 2017). Notwithstanding the informative theoretical value of evidence provided through such rigorous experiments, the degree to which they characterize or reflect real-world proficiency in facial identity processing remains unknown (Ramon et al., 2019a, 2019b).

Indeed, other tests have been designed with the intention of assessing behavior that is pertinent to real life. One frequently used paradigm involves simultaneous identity matching in a two-alternative forced-choice (2AFC) scenario. This is thought to parallel identity verification at, for example, a passport control point where naturally occurring variation in ambient images is known to affect performance (Burton et al., 1999; Megreya & Burton, 2006). Unfortunately, some of the tests used to identify SRs (e.g., the GFMT) lack sensitivity, making them inappropriate to identify highly proficient face processing abilities (Bobak et al., 2016b; Ramon et al., 2019a, 2019b). This situation is further exacerbated by two additional aspects. First, 2AFC paradigms, including for example the Expertise in Facial Comparison Test (EFCT) and the Person Identification Challenge Test (PICT) (White et al., 2015), have been used to further probe individual differences in face matching (Phillips et al., 2018). Although developed to assess performance under challenging situations (White et al., 2015), the EFCT and PICT involve face matching under optimal viewing conditions, in other words using full-frontal face images—a necessary requirement to meet the original goal of comparing human and machine performance (Phillips & O'Toole, 2014). Additionally, as they require observers to make simple same/different decisions, they involve a constant probability of correct responses on a trial-to-trial basis. Most importantly, however, the “pedestrian notion” of speed–accuracy trade-offs (Heitz, 2014; see also Luce, 1986) is commonly not considered; observers can obtain high test scores at the expense of prolonged RTs, which are not reported when the EFCT and PICT are used (e.g., Phillips et al., 2018; for a similar approach adopted in the context of pairwise face-matching tests see also Bobak et al, 2016c; Robertson et al., 2016).

### The solution: standardization of challenging and ecological valuable laboratory tests

**Fig. 1:**
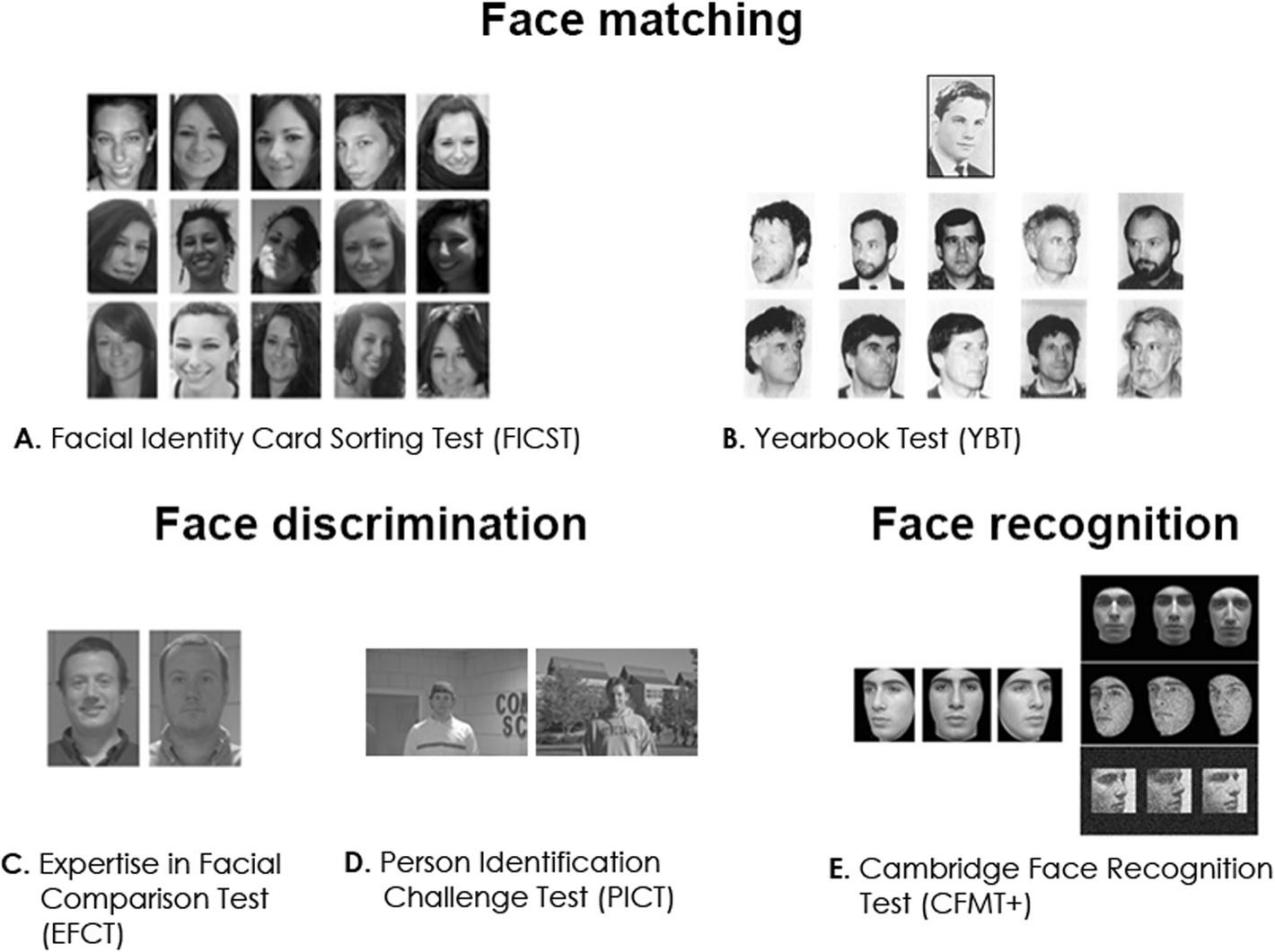
Examples of stimulus material presented in the tests of face processing administered in this study. Observers completed tests of face matching involving naturally occurring **a** image-related (FICST; Jenkins et al., 2011), or **b** aging-related variations (YBT; Bruck, Cavanagh, & Ceci, 1991). Additional measures included previously established tests of **c**, **d** face discrimination (EFCT and PICT; White et al., 2015), and **e** recognition (CFMT+; Russell et al., 2009). Note that these illustrations are for demonstration purposes and may differ from the stimulus material used (detailed descriptions are provided in the methods section). Consent/rights for publication obtained for individuals depicted

In the context of identifying individuals that could provide a substantial contribution in applied settings, a growing body of literature has expressed the need for more ecological and *challenging* assessment of face processing abilities (Bate et al., 2018, 2019; Lander, Bruce, & Bindemann, 2018; Moreton et al., 2019; Ramon et al., 2019a; Robertson & Bindemann, 2019). We aim to contribute to filling this void concerning the assessment of facial identity processing under both realistic and challenging conditions. To this end, we tested a large group of individuals of all ages (*N* = 252) with previously reported tests of facial identity matching. These tests tap into invariance of facial representations by measuring facial identity matching ability across two dimensions that are pertinent to real life challenges. The YBT (for examples see Fig. 1b) captures identity matching across significant age-dependent changes in facial appearance (i.e., 25 years; Bruck et al., 1991). The FICST (see Fig. 1a) probes the ability of telling together and telling apart identities across superficial image variations (lighting, make-up, hairstyle, etc.; Andrews, Jenkins, Cursiter, & Burton, 2015; Jenkins et al., 2011). Because of the nature of the tasks and face stimuli, these two tests mimic real-world challenges in face processing. The YBT, for instance, resembles the situation of encountering acquaintances or friends after considerable time periods. In a policing context, it might translate into scenarios where comparison images of alleged criminals in a line-up are dated and experts or witnesses are required to disregard age-related changes. The task and face stimuli used in the FICST, on the other hand, could resemble a situation where police officers are required to determine whether footage from multiple crime scenes depicts the same individual, or whether image material viewed in the context of child abduction or abuse depicts the same victim(s). In this case, face processing would not only be challenging due to variation in image quality, but also due to potential disguises, or time periods between image acquisitions (Megreya, Sandford, & Burton, 2013). Additionally, image ambiance is inherently larger in the FICST due to the presence of 20 images per identity (versus two in the PICT and EFCT), and in both the FICST and YBT faces are depicted in greyscale and from different viewpoints (as opposed to full-frontal color images in the EFCT and PICT; see Fig. 1). Note that the EFCT and PICT stimuli were presented in color. Finally, instead of requiring simple same/different decisions between two faces as in the EFCT and PICT, the FICST and YBT involve a substantially greater number of possible responses. In the FICST, participants are blind to the number of identities portrayed in the 40 pictures they are supposed to sort. In the YBT, observers have to match five target pictures portraying young adults with the corresponding five probe images, which are presented along with five distractors (Bruck et al., 1991). Consequently, the FICST and YBT include a broader decisional space, which has been experimentally shown to increase task difficulty (Ramon, Sokhn, & Caldara, 2019; Ramon, Sokhn, Lao, & Caldara, 2018), and they also resemble more challenging real-world situations beyond identity verification.

In light of the aforementioned considerations, the goal of this study was twofold. We aimed to provide normative data for the YBT and FICST, which in our opinion are more ecologically meaningful and challenging tests of unfamiliar face matching (i.e., perception) than the EFCT and PICT. Beyond this, we sought to determine the relationship between performance measures provided by the YBT and FICST, as well as the EFCT and PICT, with the most commonly used tool to assess face cognition, the long form of the Cambridge Face Memory Test (CFMT+; Russell et al., 2009). Note that, although the CFMT+ represents a test of face *recognition*, it remains the most commonly used means to identify superior face processing skills (cf. Bobak et al, 2016b; Noyes, Phillips, & O'Toole, 2017; Ramon et al., 2019a).

To anticipate our findings, and in line with previous work, our results demonstrate that facial identity matching is considerably impacted by superficial image variations and age-related changes in appearance. Importantly, across all tests of face perception reported, which rely on simultaneous matching of facial identity displayed in natural images (i.e., involve no memory component), the EFCT and PICT were the least challenging. Based on these observations, we advocate for increased use of more challenging measures that involve manipulations that are pertinent to real-life settings, such as the YBT and FICST. Compared to 2AFC scenarios, in these tests time-consuming strategies are less effective for achieving high-performance accuracy. The conditions under which tools are developed (cf. EFCT and PICT; probing face matching under ideal visual conditions as required per dated automatic face processing solutions) have to be carefully considered in combination with the real-life roles in which they are deployed (for example, identity verification encountered by passport control officers, doormen, or cashiers).

## Methods

### Participants

A total of 252 observers (150 females; age range 18–80 years) completed the aforementioned YBT. Of this cohort, 218 additionally completed the FICST. In addition to the YBT and FICST, 181 observers completed the CFMT+, EFCT, PICT, and Cambridge Face Perception Test (CFPT). Detailed demographic information for each sample is provided in Table 1. This entire cohort included (under)graduate students from the University of Fribourg who received course credit for participation as well as individuals from a wide range of professional fields and with varying levels of education (family, friends, colleagues and acquaintances of the experimenters) who participated voluntarily without compensation. All procedures were approved by the local Ethics Committee and conducted following the tenets of the Declaration of Helsinki (Puri, Suresh, Gogtay, & Thatte, 2009). Written informed consent was obtained from each participant.

**Table 1:** Demographic information for all samples reported

YBT only	
Sample size (all/male/female)	*N* = 252/101/151
Handedness (right/left/ambidextrous)	224/26/2
Mean age (SD), range (years)	29.0 (12.7), 18–80
FICST only	
Sample size (all/male/female)	*N* = 218/98/120
Handedness (right/left/ambidextrous)	195/21/2
Mean age (SD), range (years)	29.8 (13.1), 18–80
All tests	
Sample size (all/male/female)	*N* = 181/78/103
Handedness (right/left/ambidextrous)	161/19/1
Mean age (SD), range (years)	29.2 (13.1), 18–80

*FICST* Facial Identity Card Sorting Test, *SD* standard deviation, *YBT* Yearbook Test

### Materials and procedures

Each observer completed the paper-based YBT; if participation was not terminated, additional tests were administered. These included the paper-based FICST, as well as four computer-based tests (CFMT+, EFCT, PICT and CFPT) which were completed (in randomized order) using the standalone Java applications provided by the authors of the original studies. Performance measures were recorded in keeping with previously adopted procedures, or involved additional measures (e.g., RT registration) as detailed below. Examples of stimulus material presented in each experiment are displayed in Fig. 1. Note that since, currently, the YBT and FICST are relatively infrequently used, we provide a list of aspects requiring careful consideration for appropriate application.

### Tests of face perception standardized in this study

#### Yearbook Test

This test of face perception involves the original material reported in Bruck et al. (1991), which differs from the demonstration provided in Fig. 1. The YBT was originally designed to test recognition and identification of personally familiar faces (former classmates from high school) 25 years later at a high school reunion (Bruck et al., 1991). We used this test as a measure of simultaneous unfamiliar face matching across age-related changes in facial appearance. The material consists of eight pages portraying images of same-gendered individuals. On each page, five (younger) target identities are depicted on the left, along with 10 probe images on the right (see Fig. 1b). Probe images comprised the five target and foil identities of the same age (i.e., all 25 years older than target identities), respectively. Images (220 × 350 mm) included variations in viewpoint and depicted paraphernalia and external cues. Contrary to the original study (Bruck et al., 1991), none of the participants were familiar with any of the depicted identities. Therefore, participany4csxts were instructed to neglect the middle columns designed for familiar face recognition and identification, and to simply indicate (by providing a number) which of the probe images corresponded to a given target face. For each observer, we report the raw score of correctly matched identities. Note that only 35 items could be analyzed as the remaining five were not identifiable by the authors of the YBT (personal communication, Bruck & Cavanaugh, 2018).

#### Facial Identity Card Sorting Test

This face perception test involves a simultaneous card-sorting procedure as reported by Jenkins et al. (2011), and represents a measure of face matching across image-related variation in facial appearance. A selection of 40 images, half of which depict one of two Dutch celebrities (Chantel Janzen and Bridget Maasland), kindly provided by Jenkins and Burton (personal communication, 2017) were used as stimulus material (multiple sets of 40 laminated greyscale images sized 38 x 50 mm). The twenty ‘ambient images’ (Jenkins et al., 2011; Sutherland et al., 2013) of each individual entailed natural face variability that occurs in real life as they were taken from publicly available photographs of each celebrity found on the internet. When instructed to group 40 such ambient images by the number of perceived identities, observers who are unfamiliar with the depicted identities report perceiving a median of 7.5 identities (mode 9; range 3–16; Jenkins et al., 2011; see also Andrews et al., 2015). Here, the 40 image cards were randomly positioned (in upright orientation) in front of observers who had unlimited time to group them according to the number of identities they subjectively perceived as distinct, with each group of images representing a single individual (for an example of similar image variations, see Fig. 1a). The experiment ended if observers spontaneously and correctly grouped the images correctly into two categories. However, if observers formed a different number of groups, they completed a second sorting phase. In phase 2, the cards were again mixed and spread out randomly; however, observers were now instructed to separate the 40 images into two groups. The sorting responses of the observers were photographed; for each phase, the number of piles and total number of errors was registered (note that an identity match was considered erroneous if it did not correspond to a given group’s predominant identity). Analyses were performed on a composite score calculated based on both measures obtained during the first sorting phase (see below).

### Optimal YBT and FICST application: methodological considerations

Based on our experience, we point out three aspects that require careful consideration to ensure the most efficient and informative application of the YBT and FICST. The first pertains to the number of trials contained in the YBT, relative to the number of currently analyzable trials. As mentioned, the authors of this test were able to provide the correct responses for 35 of the total of 40 items. Currently, efforts are underway to determine the most probable correct responses for the missing items using a wisdom of crowd approach (cf. Balsdon, Summersby, Kemp, & White, 2018; Phillips et al., 2018) by testing a large number of high-performing individuals to ensure maximal validity of such data-driven estimates. Once generated, the normative data provided here can be re-evaluated to include responses for the currently not considered items, and the test could be employed in two versions.

The second consideration relates to application of the FICST. Naturally, it is imperative that observers have no prior information regarding the number of identities displayed, in other words they should not have participated in an experiment with a similar procedural setup. As evident from the data reported for phase 2, observers are clearly more proficient when provided with top-down information regarding the decisional space in which identity matches are to be provided (cf. Ramon, 2018a; Ramon et al., 2018; Ramon et al., 2019c). Relatedly, attention must also be paid the possibility of observers being familiar with the two Dutch celebrities depicted. Such knowledge would serve as an effective prior facilitating identity grouping (see Andrews et al., 2015; Jenkins et al., 2011). Even if different versions of this test were created in the future that involved different set sizes with varied numbers of individuals depicted, prior completion or knowledge of the original FICST may decrease the initially observed effect of identity separation and grouping reported here and elsewhere (Andrews et al., 2015; Jenkins et al., 2011).

A final, seemingly trivial, consideration concerns the quality of image material used both for the YBT and FICST. As noted, we were kindly provided with a physical copy of the original YBT used by Bruck et al. (1991); a large number of digital image copies of both Dutch celebrities were provided to recreate a version akin to the FICST reported by Jenkins et al. (2011). We observed quality and appearance variations in the reproduction and duplication process. To ensure that observers were confronted with identical image material, we opted for use of the FICST material (printed and laminated for repeated usage) and reproduced the YBT based on its scanned version using the same printer (and printer settings). Researchers interested in using this material should contact the corresponding author to receive a replica to be used without further reproduction until the potential impact of reproduction-related quality variations on observers’ performance is established.

### Commonly reported tests of face recognition and perception included in this study

#### Long form of the Cambridge Face Memory Test

This computer-based test was developed by Russell et al. (2009) with the aim of assessing face recognition for experimentally learned identities. This computer-based test comprises increasingly difficult parts (see Fig. 1e), in which grayscale cropped male face stimuli are presented (6 target identities, 46 distractors). Participants encode images of target identities from different viewpoints before subsequently selecting the target identity among two distractors. As this test progresses, the trials become increasingly more difficult, as illumination, orientation and information availability are manipulated (see Fig. 1e). For each observer, we recorded the number of correct responses obtained across the total of 102 items of the CFMT+.

#### Expertise in Facial Comparison Test

This test of face perception was reported by White et al. (2015) and assesses face discrimination ability measured in the context of a computer-based simultaneous matching task. Half of the (total of 84) trials depict the same identity; the remainder involve pairs of identities that were deliberately “similar looking” (White et al., 2015). To better approximate its more recent application (cf. Noyes et al., 2018; Phillips et al., 2018), observers provided binary same/different responses for color uncropped faces with varied facial expressions and on different backgrounds (see Fig. 1c), which were presented for a maximum of 30 s (versus 5 or 7 scale responses, and 2-s presentation durations; cf. White et al., 2015).^1^ For each observer, we recorded accuracy and RTs. As discussed below (see “Analyses”), we deem consideration of both accuracy and RTs at the individual level (as opposed to merely the group level; e.g., White et al., 2017) especially relevant when aiming to provide normative data from a large and heterogeneous sample against which individual performance can be reliably compared.

#### Person Identification Challenge Test

This test of face perception involves the same procedure and experimental settings, and hence process, as the EFCT (White et al., 2015). The only difference is that the face stimuli portray individuals from a greater distance, and therefore display more external cues and environmental information. This test involved the same testing procedure (presentation duration, responses registered) as described above for the EFCT.

In addition to these three frequently applied tests, and as mentioned above, observers also completed the CFPT (Duchaine, Germine, & Nakayama, 2007), a test of face perception that involves sorting upright and inverted faces according to their similarity with a simultaneously presented target face. This test was not intended to be considered in our analyses; it was employed to enable our students to independently formulate and probe hypotheses using the data from the total database.

### Analyses

#### The need for computation of composite scores

To take into account the behavioral measures registered for a given test, composite performance scores were calculated for all tests with the exception of the YBT and CFMT+, for which only raw scores (i.e., number of correctly matched or recognized faces) were considered.

For the FICST, perfect performance involves separating the 40 images into two piles without inclusion errors. While previous studies considered only the number of piles as an index of performance (Andrews et al., 2015; Jenkins et al., 2011), here we calculated a composite score to consider both possible sources of errors (inclusion and separation). This allows distinction between observers, who randomly versus accurately create two identity piles. Any number of piles exceeding two was considered as an additional mistake(s). The FICST scores of the observers were computed by subtracting two from the sum of piles and errors. Thus, a score of zero corresponds to perfect performance; the higher the FICST score, the less proficient an observer’s performance for grouping images by identity.

Note that for the YBT, FICST and CFMT+, RTs were not considered as informative for the following reasons. First, for the YBT and FICST, provided responses are not based on discrete pairs. Moreover, identity match decisions can be changed. Furthermore, completed identity matches (correct or incorrect) determine the nature of subsequent responses. For the CFMT+, the presentation of the to-be-learned faces is fixed, while recognition decisions provided for the test face trio is unlimited. As in this context performance is determined by encoding proficiency and recognition ability, longer decision times will not facilitate recognition performance, and hence no systematic speed–accuracy trade-off is expected.

From a procedural perspective, the EFCT and PICT are the least complex compared to the YBT, FICST and CFMT+. Both require binary, perceptually based decisions for face stimuli presented simultaneously without time constraints. In such an experimental context, we reasoned that consideration of RTs is imperative. Under simultaneous matching conditions observers are more likely to display speed–accuracy trade-offs compared to, for example, recognition tasks. Even highly impaired prosopagnosic individuals can achieve accurate performance at the expense of vastly prolonged RTs, which is why it is common practice to take into account RTs in studies of both healthy and impaired individuals (e.g., Behrmann, Avidan, Marotta, & Kimchi, 2005; Biotti et al., 2017; Lao, Vizioli, & Caldara, 2013; Marotta et al., 2002; Tarr, 2003; White et al., 2017). Indeed, according to many, “the notion of speed–accuracy tradeoff (SAT) is pedestrian” (Heitz, 2014; see also Luce, 1986).

The highly heterogeneous distribution of RTs observed for the EFCT and PICT supports this notion and suggests that many healthy observers may indeed show speed–accuracy trade-offs. Leaving aside the implications for studies that did not consider RTs and therefore neglect the impact of speed–accuracy trade-offs (e.g., Bobak et al., 2016a; Bobak et al., 2016b; Bobak et al., 2016c; Burton et al., 2010; Noyes et al., 2018; Phillips et al., 2018; Robertson et al., 2016; White et al., 2015), here we opted to consider both speed and accuracy by computing the inverse efficiency (IE) score of the observers (Townsend & Ashby, 1978). This is crucial given our goal of providing normative data against which individual performance can be compared in order to determine potentially superior performance.

### Statistics

#### Behavioral measures considered

Behavioral responses from all five tests were subject to descriptive and correlation analyses. Descriptive statistics are provided separately for all measured behavioral responses, as well as composite scores. Correlation analyses were performed on the YBT and CFMT+ raw scores, and (following the aforementioned sources of errors and potential speed–accuracy trade-offs) the FICST, EFCT and PICT composite scores.

#### Analyses of high-performing individuals

Additionally, in order to determine the predictive value of a given test, we investigated how the top performers on each test scored on the remaining ones. First, and independently for each of the five tests, we identified those participants whose scores were located in the top 5% (*n* = 9). We then computed each subsample’s mean performance and retained the value as a reference average. Finally, we extracted the score of each subsample for each respective other test and compared the mean to each test’s reference average.

## Results

### Descriptive analyses

#### Normative data for the Yearbook Test

**Fig. 2:**
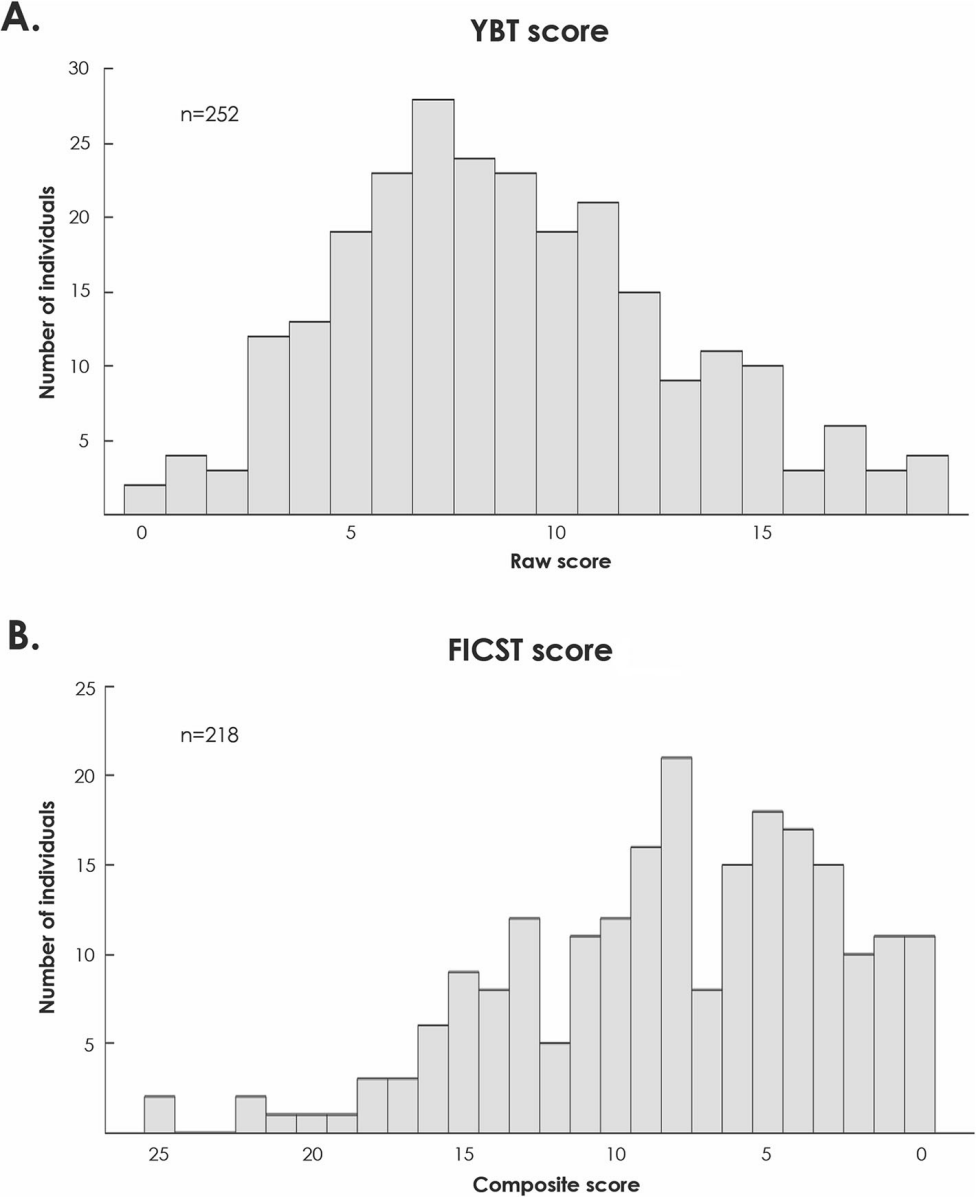
Distribution of scores obtained for the **a** Yearbook Test (YBT) and **b** Facial Identity Card Sorting Test (FICST). Across both tasks, low and high scores are represented on the left and right, respectively

The normative data collected from 252 observers are reported in Table 2 and visualized in Fig. 2a. On average, observers correctly matched 8.8 ± 4.0 (median 8.0) of the 35 analyzable items (see “Methods”).

**Table 2:** Normative data—number of correct identities achieved in the Yearbook Test

	All participants (*N* = 252)
Mean (SD)	8.8 (4.0)
Median	8

*SD* standard deviation

#### Normative data for the Facial Identity Card Sorting Test

The normative data obtained from 218 participants are reported in Table 3 and visualized in Fig. 2b. On average, during phase 1 observers perceived 7.6 ± 4.9 (median 7) identities and made 2.4 ± 3.3 (median 1) errors. In phase 2, the average number of errors was 3.4 ± 4.4 (median 2).

**Table 3:** Normative data—numbers of piles and errors expressed in the Facial Identity Card Sorting Test (FICST)

	All participants (*N* = 218)
Phase 1	
Number of piles	
Mean (SD)	7.6 (4.9)
Median	7
Total number of errors	
Mean (SD)	2.4 (3.3)
Median	1
FICST score	
Mean (SD)	8.0 (5.3)
Median	8.0
Phase 2	
Total number of errors	
Mean (SD)	3.4 (4.4)
Median	2

Note that the FICST scores computed for phase 1 were considered in the subsequent analyses

*SD* standard deviation

#### Normative data for observers who participated in all five tests

**Fig. 3:**
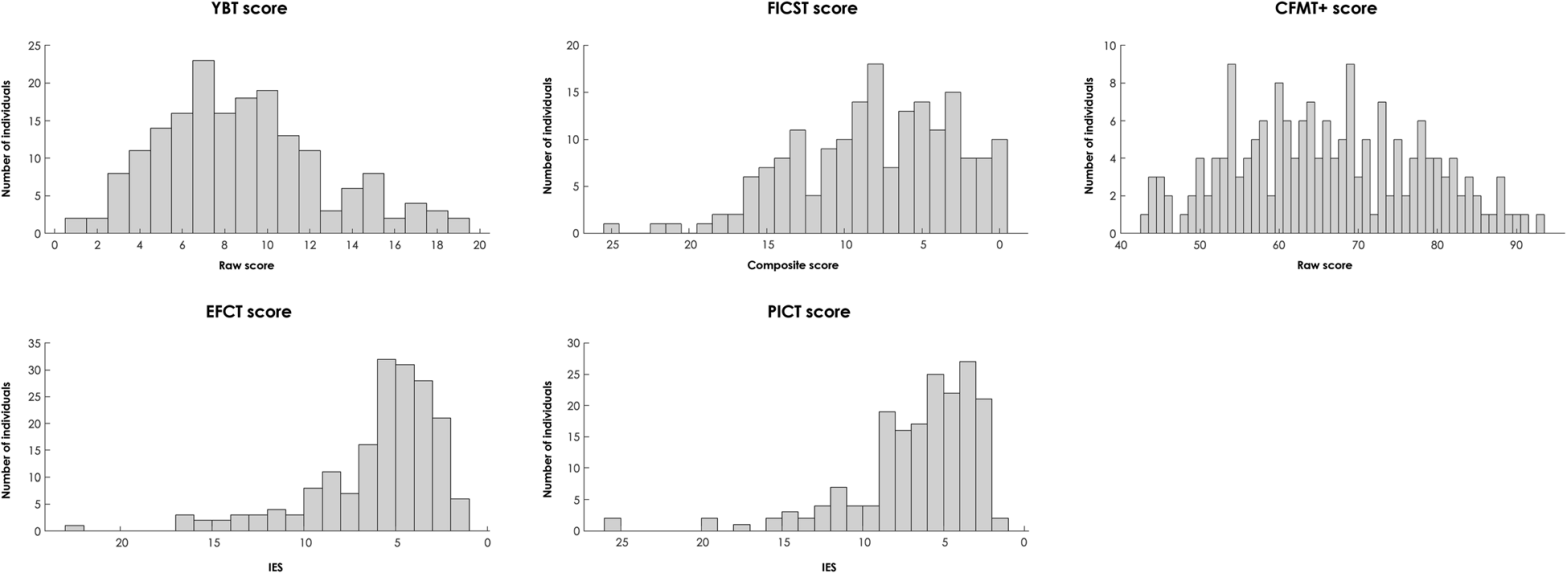
Distributions of scores obtained for observers who participated in the Yearbook Test (YBT), Facial Identity Card Sorting Test (FICST), long form of the Cambridge Face Memory Test (CFMT+), Expertise in Facial Comparison Test (EFCT) and Person Identification Challenge Test (PICT). Note that scores representing low and high performance are represented on the left and right of the *x* axes, respectively (i.e., high inverse efficiency scores (IES) for the EFCT and PICT indicate low performance); for visualization purposes one extreme value was removed for the CFMT+ and PICT, respectively

Figure 3 and Table 4 summarize the normative data obtained for the subsample of 181 observers who participated in all tests (YBT, FICST, CFMT+, EFCT and PICT). Mirroring the observations made for the larger sample (see Table 1), in the YBT observers correctly matched 8.8 ± 3.9 (median 8.0) of the 35 items. For the FICST, as in the larger sample (see Table 2), in phase 1 observers perceived on average 7.4 ± 4.6 (median 7) identities and made 2.5 ± 3.4 (median 1) errors; in phase 2 they made on average 3.4 ± 4.4 (median 2) errors. For the CFMT+, observers achieved an average raw score of 66 (median 65). The results for the EFCT and PICT (see Table 4) were similar, although accuracy scores were higher for the EFCT than the PICT, leading to comparatively higher IE scores (i.e., inferior performance) for the PICT as compared to the EFCT.

**Table 4:** Normative data for participants who completed the YBT, FICST, CFMT+, EFCT and PICT

	All participants (*N* = 181)
YBT	
Mean (SD)	8.8 (3.9)
Median	8
FICST	
Number of piles	
Mean (SD)	7.4 (4.6)
Median	7
Total number of errors	
Mean (SD)	2.5 (3.4)
Median	1
FICST score	
Mean (SD)	8.0 (5.1)
Median	8
Phase 2	
Total number of errors	
Mean (SD)	3.4 (4.4)
Median	2
CFMT+ raw score	
Mean (SD)	65.9 (11.9)
Median	65
EFCT	
Mean accuracy in % (SD)	77.9 (8.2)
Median accuracy	78.6
Mean RT in s (SD)	4.7 (2.7)
Median RT in s	4.0
EFCT inverse efficiency score	
Mean (SD)	6.0 (3.5)
Median	5.1
PICT	
Mean accuracy in % (SD)	72.8 (11.0)
Median accuracy	72.5
Mean RT in s (SD)	4.9 (3)
Median RT in s	4.1
PICT inverse efficiency score	
Mean (SD)	6.9 (5.1)
Median	5.8

*CFMT+* long form of the Cambridge Face Memory Test, *EFCT* Expertise in Facial Comparison Test, *FICST* Facial Identity Card Sorting Test, *PICT* Person Identification Challenge Test, *RT* reaction time, *SD* standard deviation, *YBT* Yearbook Test

### Correlation analyses

**Fig. 4:**
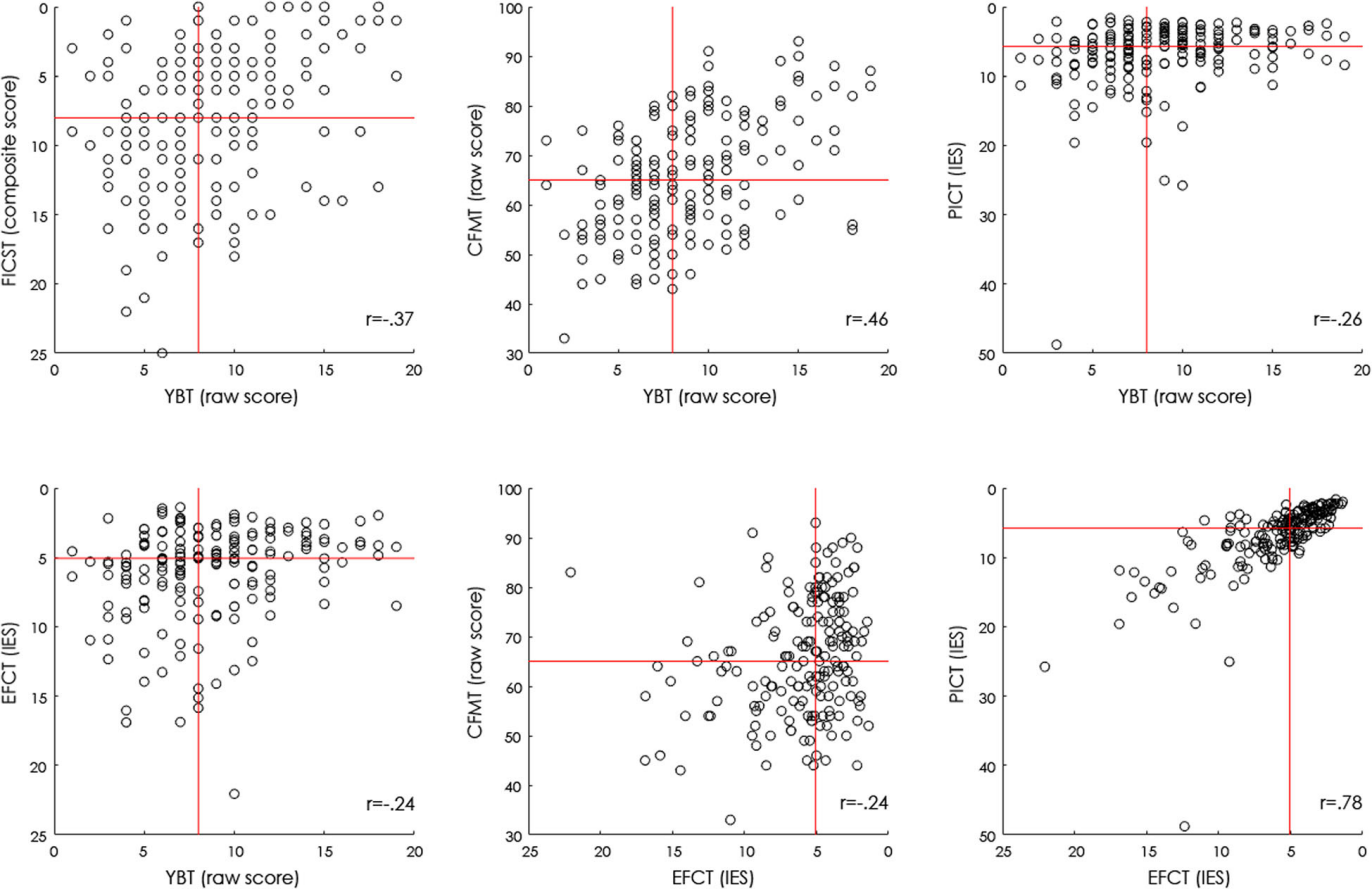
Significant correlations between measures. Visualized here are the significant correlations (see Table 5) between the Yearbook Test (YBT) and Facial Identity Card Sorting Test (FICST), long form of the Cambridge Face Memory Test (CFMT+), Person Identification Challenge Test (PICT) and Expertise in Facial Comparison Test (EFCT) (top row to bottom left), as well as EFCT and CFMT+ (bottom middle), and PICT (bottom right), respectively. Red lines indicate the median of each test. IES inverse efficiency score

Table 5 summarizes the results of Spearman correlations computed between the five tests, which are visualized in Fig. 4. These analyses revealed the following significant relationships (Bonferroni-corrected for multiple comparisons). First, we observed a significant positive relationship between the YBT and CFMT+ raw scores; greater numbers of correctly matched identities in the YBT were associated with more items recognized in the CFMT+. Second, a positive relationship also emerged between the IE scores obtained for EFCT and PICT; better EFCT performance (i.e., lower IE scores) was associated with better PICT performance. Third, we found a significant negative relationship between YBT and FICST scores, as well as between the YBT and PICT, and YBT and EFCT IE scores. These correlations indicate that greater numbers of correctly matched YBT items were associated with both less errors and piles in the FICST, and better performance for the PICT and EFCT. Finally, EFCT IE scores also correlated negatively with CFMT+ raw scores, indicating that higher CFMT+ performance was associated with more proficient EFCT performance. None of the remaining relationships reached significance.

**Table 5:** Correlation coefficients computed for the CFMT+, YBT, FICST, EFCT and PICT scores

	CFMT+	YBT	FICST	PICT	EFCT
CFMT+		.46*	−.19	−.10	−.24*
YBT			−.37*	−.26*	−.24*
FICST				.06	.12
PICT					.78*
EFCT					

Asterisks indicate significant correlations (Bonferroni-corrected; *p < .005*); note that for the FICST, PICT and EFCT lower values indicate better performance (see “Methods” section)

*CFMT+* long form of the Cambridge Face Memory Test, *EFCT* Expertise in Facial Comparison Test, *FICST* Facial Identity Card Sorting Test, *PICT* Person Identification Challenge Test, *YBT* Yearbook Test

### Analyses of top performing individuals

**Fig. 5:**
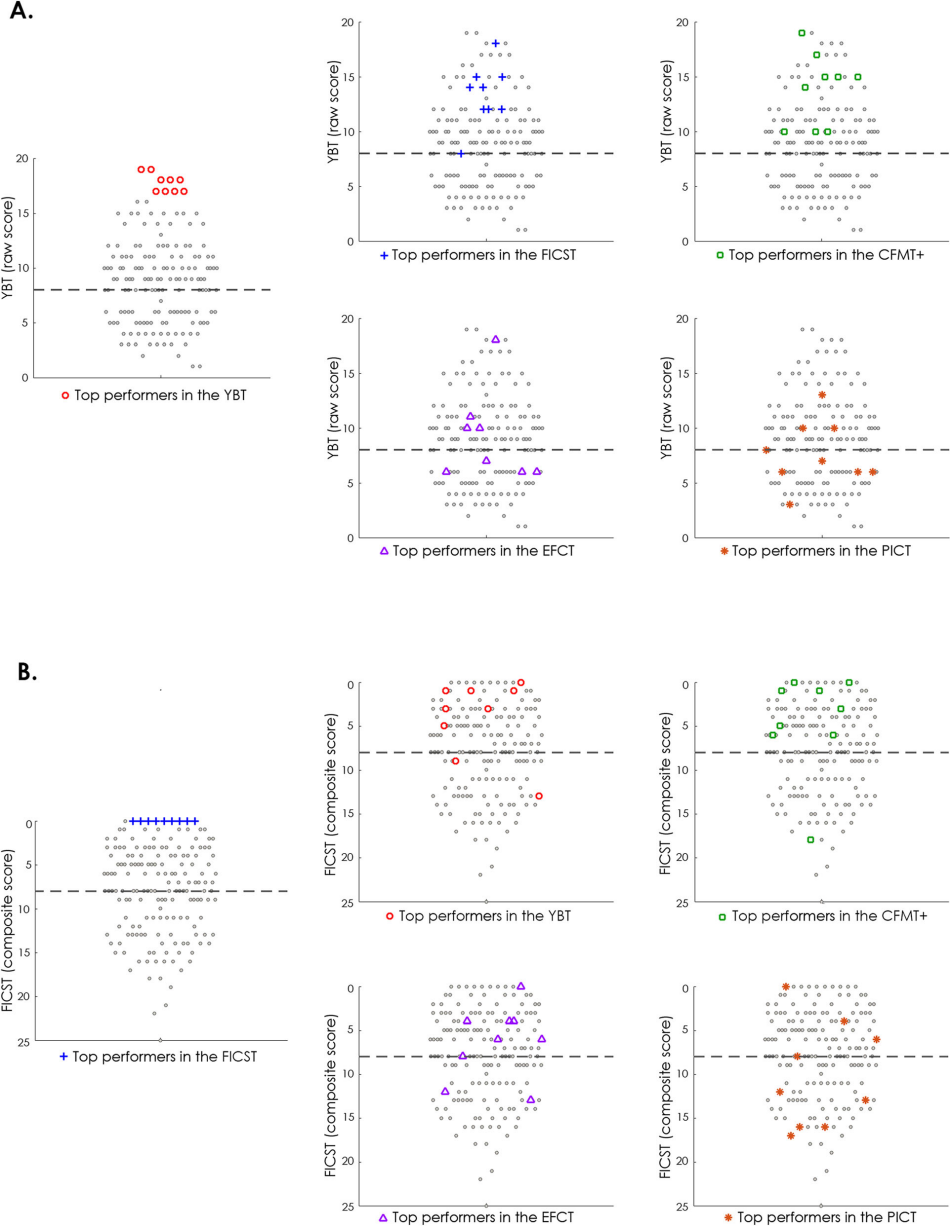
Yearbook Test (YBT) and Facial Identity Card Sorting Test (FICST) scores of the top performers identified independently per test. Visualized here are performance levels of individuals identified as the top performers (colored symbols) for the YBT (red circles), FICST (blue crosses), long form of the Cambridge Face Memory Test (CFMT+) (green squares), Expertise in Facial Comparison Test (EFCT) (purple triangles) and Person Identification Challenge Test (PICT) (orange asterisks), and their relative location among observations made (grey dots) for the **a** YBT and **b** FICST. High performers identified based on the CFMT+, YBT and FICST are more consistently located above the YBT and FICST median (black dashed horizontal line), thereby exhibiting better performance than individuals identified as top performers based on the EFCT and PICT

Table 6 summarizes the following: 1) reference scores computed across the top 5% performing individuals identified independently per test (*n* = 9); 2) their respective performance on the remaining tests; and 3) the mean and standard deviation for each test based on the normative sample. For the YBT, high-performing individuals identified via the CFMT+ and FICST performed more similarly to the reference score compared to the top performers identified via the EFCT and PICT (Table 6, Fig. 5a). Moreover, CFMT+ and FICST high performers obtained an average YBT score that was one standard deviation above the mean of the normative sample. Focusing on FICST performance patterns, the means of the top performers on CFMT+ and YBT were more similar to the reference mean of the top performers on FICST compared to those of the high performers on EFCT and PICT (Table 6, Fig. 5b). Additionally, with respect to the FICST normative average, top performers on CFMT+, YBT and EFCT scored better, but within one standard deviation of the mean. Focusing on the CFMT+, YBT and FICST, high performers scored above the normative average and more similarly to the reference mean of the top performers on CFMT+. This was contrasted by the top performers on PICT and EFCT, who also exhibited average performance that was below the CFMT+ normative average. Finally, top performers identified for the EFCT and PICT performed better on these tests than the normative sample and more similarly to the reference mean of the top performers on these tests than high performing individuals identified via the CFMT+, YBT and FICST (Additional file 1: Figure S1).

**Table 6:** Top performers mean performance for each of the five administered tests

	Top 5% performers					Mean ± standard deviation of the normative data (*n* = 181)	Median of the normative data (*n* = 181)
	CFMT+	YBT	FICST	EFCT	PICT		
CFMT+	**88.9**	75.8	75	64.1	64.8	65.9 ± 11.9	65
YBT	13.9	**17.8**	13.3	9.0	7.7	8.8 ± 3.9	8
FICST	4.4	4.0	**0.0**	6.3	10.2	8.0 ± 5.1	8
EFCT	4.9	4.2	5.2	**1.9**	2.0	6.0 ± 3.5	5.1
PICT	5.9	4.8	6.3	2.4	**2.2**	6.9 ± 5.1	5.8

Reference mean scores computed for the top performers of a given test are shown in bold

*CFMT+* long form of the Cambridge Face Memory Test, *EFCT* Expertise in Facial Comparison Test, *FICST* Facial Identity Card Sorting Test, *PICT* Person Identification Challenge Test, *YBT* Yearbook Test

## Discussion

Despite decades of research, the ability of humans to process facial identity continues to attract considerable attention. While group studies have previously dominated the field, more recently an increasing interest in individual differences has emerged, both at the behavioral and neural level (cf. Bruce et al., 2018; Stacchi, Liu-Shuang, Ramon, & Caldara, 2019). A growing body of work has focused on identifying and characterizing individuals with remarkable face processing abilities, the so-called SRs (Ramon et al., 2019a, 2019b; Russell et al., 2009). Here, we aimed to contribute to advancing knowledge of this population by standardizing two procedurally simple but highly challenging behavioral face perception tasks. Both involve two ecologically relevant aspects of face cognition by probing invariant facial identity matching across naturally occurring conditions that are commonly neglected—aging and image ambiance. These tests are promising solutions to fill the current void regarding highly sensitive applied screening tests suitable for identifying individuals with superior face processing skills (see also Ramon et al., 2019a, 2019b).

### The YBT and FICST as novel face perception tests to identify superior face processing

Arguably, one of the most commonly employed measures of face processing is the CFMT+ (Russell et al., 2009). This test of face recognition, in which high performance can result from superior face perception or memory, has been frequently deployed to identify individuals with superior face processing abilities (e.g. Bobak et al., 2016; Noyes et al., 2017; Ramon et al., 2019a). Face matching tests created as alternative assessment tools for face perception (e.g., the GFMT; Burton et al., 2010; White et al., 2015) unfortunately often lack sensitivity (Bate et al., 2018; Bobak et al., 2016; Fysh, 2018; Fysh & Bindemann, 2018) or do not consider potential speed–accuracy trade-offs, which are particularly relevant when observers have unlimited processing time to perform simple binary decisions. Such procedural aspects are often neglected, and there is currently no accepted “gold standard test” of superior face processing ability (Ramon et al., 2019a, 2019b).

In light of the shortcoming of tests currently used to identify individuals with superior face perception, here we aimed to provide normative data for the YBT (Bruck et al., 1991) and the FICST (Jenkins et al., 2011). We deem both to be ecologically meaningful and challenging tests of face perception which are not affected by the aforementioned procedural considerations. The YBT assesses unfamiliar face matching across substantial age-related changes, while the FICST demonstrates the effect of superficial or ambient image changes on perception of facial identity (see Fig. 1). Both tests were standardized on a large and heterogeneous cohort. To promote their respective usage, the data for all cohorts and tests reported here are made publicly available on the public repository Open Science Framework (https://osf.io/kagtr/).

A comparable number of observers achieved perfect scores for the FICST and fell within the 95th percentile for the YBT, respectively. However, for the YBT, no observer achieved the highest possible score of 35 correct identity matches. Thus, the ecologically relevant and challenging face matching tests standardized here are characterized by higher task difficulty than tests that have previously been employed as “standard screening” (Noyes et al., 2018) tools, despite their aforementioned lack of sensitivity in both healthy observers and individuals with developmental prosopagnosia (e.g., White et al., 2017). Conversely, floor effects, which are a common problem when aiming to calibrate challenging tasks suitable for identifying high performance (cf. Crowds matching test; Bate et al., 2018), were found for neither the YBT nor FICST. Thus, we believe that these tests provide much needed alternatives of unfamiliar face matching that are easy to use across varied contexts and in pursuit of the goal of identifying superior face perception skills.

### Evaluation of the YBT and FICST as predictors of face processing skill: comparison with CFMT+, EFCT and PICT

To determine the relationship with previously deployed tests of face cognition, the YBT and FICST were compared with the most commonly used measure of face recognition (CFMT+; Russell et al., 2009) and two frequently reported tests of face perception (the EFCT and PICT; White et al., 2015). As used recently, here the EFCT and PICT were employed under unconstrained viewing conditions (Noyes et al., 2018; Phillips et al., 2018). The majority of studies involving the latter face matching tests have focused exclusively on accuracy scores obtained in highly homogeneous and/or small samples of young observers, but did not consider RTs (e.g., Bobak et al., 2016; Noyes et al., 2018; Phillips et al., 2018; Robertson et al., 2016). On the contrary, here we considered both accuracy and RTs. Aiming to account for the “pedestrian notion” of speed–accuracy trade-offs (Heitz, 2014; see also Luce, 1986), which can be found in healthy as well as in abnormal populations (Behrmann et al., 2005; Biotti et al., 2017; Geskin & Behrmann, 2018; Herzmann, Danthiir, Schacht, Sommer, & Wilhelm, 2008; Marotta et al., 2002; Ramon et al., 2010; Ramon & Rossion, 2010; Tarr, 2003; White et al., 2017), and which may vary across age (Hildebrandt, Herzmann, Sommer, & Wilhelm, 2010), analyses were performed on composite scores comprising both measures. Thus, alongside providing normative data for the YBT and FICST, their respective value was assessed relative to tests that have been previously used as “standard screening” tests (Bobak et al., 2016; Noyes et al., 2018) of individual face processing skills.

As noted, the distribution of data observed for the YBT and FICST demonstrate that they indeed provide challenging tools to assess unfamiliar facial identity matching. Interestingly, the highest correlation was found between the YBT and the procedurally distinct face recognition test CFMT+ (Russell et al., 2009), and the YBT and FICST. Weaker correlations emerged between the YBT and PICT, and YBT and EFCT, respectively. On the other hand, the FICST correlated with neither the EFCT nor PICT which, likely due to their procedural similarity, correlated highly with one another.

Based on the process measured, one might expect the YBT and FICST, which both tap into face perception, to correlate more strongly than the YBT and CFMT+, which assesses face recognition. Interestingly, instead, the CFMT+ and YBT correlated more strongly than the YBT and FICST. In our opinion, these findings highlight the need to carefully consider procedural differences across tests. Specifically, which process a given test measures might not be the only element to consider. For example, despite assessing two distinct processes, the CFMT+ and YBT both require individuals to find a target identity among multiple alternatives. On the other hand, while both the FICST and YBT assess face perception, due to the incorporated manipulations they involve distinct tasks, highly different face stimuli, and fundamentally different decisional spaces (Ramon et al., 2018; Ramon et al., 2019c).

These findings strengthen the concept that face cognition is highly complex and that measurements of proficiency depend on several elements. Specifically, our results show that the YBT, FICST and CFMT+ capture different, albeit related, aspects of face processing. This makes their combined application a promising tool for initial screening of superior face processing abilities. This assessment would provide: 1) a first indication of an observer’s proficiency level; 2) the direction in which further testing should proceed; and 3) potential applied fields in which an individual may be ideally deployed.

Overall, the stronger relationship observed between YBT, FICST and CFMT+ is further supported by investigation of test scores of observers identified independently per test as top performers. High-performing individuals identified via the YBT, FICST and CFMT+ scored more similarly across these three tests than individuals identified as top performers in the EFCT and PICT (Fig. 5). This suggests that, in comparison to the YBT, the EFCT and PICT have comparatively limited value in terms of predicting individuals’ face recognition ability when speed–accuracy trade-offs are taken into account in large and heterogeneous samples. Moreover, the EFCT and PICT cannot predict invariant perception of facial identity across highly varied photographs as measured by the FICST (Jenkins et al., 2011; Sutherland et al., 2013). Finally, considering the challenges inherent to the YBT and FICST, and in contrast to the EFCT and PICT, observers are less likely to excel by using strategies that can go unnoticed when only accuracy scores are considered (e.g., Noyes et al., 2018; Phillips et al., 2018) and speed–accuracy trade-offs are not accounted for (cf.Heitz, 2014 ; Luce, 1986).

We emphasize that in order to screen for potentially superior face processing skills multiple ecologically meaningful and challenging tasks must be employed to assess distinct aspects of face processing (Bate et al., 2018; Fysh, 2018; Herzmann et al., 2008; Ramon, 2018a; Ramon et al., 2019a, 2019b). To this end, accuracy scores recorded for face matching under “best-case scenarios” (Fysh, 2018) are insufficient, including, for example, moderate time periods between image creation, or the use of well-lit frontal faces as required for reliable computer-based identity matching (Phillips, Yates, Beveridge, & Givens, 2017; Phillips et al., 2018). Especially when tasks require simple same/different decisions under virtually no time constraints (e.g., 30 s here, or even 3 months in Phillips et al. (2018)), high performance accuracy cannot provide a reliable basis to identify or validate allegedly superior abilities. To maximize test sensitivity, all relevant behavioral measures obtainable (in other words accuracy and RTs) need to be considered in combination, especially in the context of procedurally simple matching tasks with unconstrained viewing conditions.

Based on our observations, we suggest that the YBT and FICST applied in combination with the CFMT+ may serve as a collection of procedurally simple screening tests of face perception and recognition. Considering the different sources of variation implemented across both tests, they may serve as powerful tools for screening of employees in applied professional settings, such as law enforcement (Ramon, 2018a; Ramon et al., 2019a, 2019b). The current findings are a step towards the expressed need for a feasible framework to assess ecologically relevant face processing skills (Moreton et al., 2019; Ramon et al., 2019a, 2019b).

### Future directions and conclusions

Having provided normative data for two challenging and ecologically meaningful tests of facial identity matching, the question is how should these measures be validated and further developed? The next steps will lie in establishing how previously reported, empirically and professionally identified SRs perform in the YBT and the FICST, and establishing how performance in the YBT and FICST relates to performance in real-life settings, such as law enforcement (Ramon, 2018a; Ramon et al., 2019a, 2019b; Ramon, 2019).

Further improvements can also be made regarding implementation of the YBT. On the one hand, a wisdom of crowds approach (cf. Balsdon et al., 2018; Phillips et al., 2018) to determine the correct responses for the currently missing five items for their subsequent inclusion will be beneficial to increase the test’s reliability. On the other hand, considering the demanding nature of this test, one fruitful endeavor is the development of a shorter screening version. To this end we have recently tested 146 individuals who completed five other previously standardized test including the CFMT+, the Kent Face Matching Test (long version; Fysh & Bindemann, 2018), FICST, 1 in 10 (Bobak et al., 2016; Bruce et al., 1999) and Models Memory Test (Bate et al., 2018), in addition to a 10-item version of the YBT (YBT-10; Fysh, Stacchi, & Ramon, in preparation; this test will be made available on request). Aligning with the data reported here for the YBT (~25% of the target identities correctly matched), observers correctly matched 3–4 items (i.e., 37%) in the abridged YBT-10.

Additionally, alternate versions of the FICST could be developed. For example, to account for sex-dependent differences in variation across ambient images, parallel male and female versions could be included, as well as other-race versions (see Tüttenberg & Wiese, 2019). As processing proficiency deteriorates with other-race faces, assessing how the ability of top performers varies across races might provide information valuable for deployment in situations that require processing of faces from varied ethnic backgrounds (e.g., specialized thefts committed almost exclusively by a specific nationality; Green, personal communication (2019)).

A final practical aspect to consider lies in developing computer-based versions of the YBT and FICST implemented as paper-based tests here. Currently, the AFC Laboratory is developing an online platform (Ramon & Docherty, in preparation) that will include, among other tests, digital versions of the YBT and FICST. This will enable screening of greater numbers of individuals or preselected cohorts, conducting item analyses, and exploiting information related to response times.

Independently of these future directions, our results emphasize the need to assess face processing abilities from various angles and considering both test-specific procedural constraints as well as the specific goal(s) of assessment. Complementing highly controlled tests, such as the CFMT+, with more realistic and challenging tasks can provide a more comprehensive and targeted understanding of observers’ abilities, and potentially higher predictive power for performance in applied settings.

## Supplementary information

**Additional file 1: Figure S1.** CFMT+, EFCT and PICT scores of the top performers identified independently per test. Visualized here are performance levels of individuals identified as the top performers (colored markers) based on the YBT (red circles), FICST (blue crosses), CFMT+ (green squares), EFCT (purple triangles), and PICT (orange asterisks), and their relative location among all observations made (grey dots) for the **a** CFMT+, **b** EFCT and **c** PICT.

## Data Availability

The datasets obtained and/or analyzed in this study will be made publicly available on the OSF. Test material used here will be made available provided permission is obtained from the original authors.

## Abbreviations

2AFC: Two-alternative forced-choice
CFMT+: Long form of the Cambridge Face Memory Test
CFPT: Cambridge Face Perception Test
EFCT: Expertise in Facial Comparison Test
FICST: Facial Identity Card Sorting Test
GFMT: Glasgow Face Matching Test
IE: Inverse efficiency
PICT: Person Identification Challenge Test
RT: Reaction time
SR: Super-recognizer
YBT: Yearbook Test

## Footnotes

1 We adopted this long presentation duration to ensure that decisions were made based on perceptual matching, and not contaminated by decisional processes or memory after stimulus offset. Additionally, we reasoned that this presentation duration exceeds the time required by healthy observers to perform this task.

## References

[CR1] Andrews S, Jenkins R, Cursiter H, Burton AM (2015). Telling faces together: Learning new faces through exposure to multiple instances. The Quarterly Journal of Experimental Psychology.

[CR2] Balsdon T, Summersby S, Kemp RI, White D (2018). Improving face identification with specialist teams. Cognitive Research: Principles and Implications.

[CR3] Barton JJS, Press DZ, Keenan JP, O’Connor M (2002). Lesions of the fusiform face area impair perception of facial configuration in prosopagnosia. Neurology.

[CR4] Bate S, Dudfield G (2019). Subjective assessment for super recognition: an evaluation of self-report methods in civilian and police participants. PeerJ.

[CR5] Bate S, Frowd C, Bennetts R, Hasshim N, Murray E, Bobak AK (2018). Applied screening tests for the detection of superior face recognition. Cognitive Research: Principles and Implications.

[CR6] Bate, S., Portch, E., Mestry, N., & Bennetts, R. J. (2019). Redefining super recognition in the real world: Skilled face or person identity recognizers? *British Journal of Psychology*. 10.1111/bjop.12392 30882903

[CR7] Behrmann M, Avidan G, Marotta JJ, Kimchi R (2005). Detailed exploration of face-related processing in congenital prosopagnosia: 1. Behavioral findings. Journal of Cognitive Neuroscience.

[CR8] Biotti F, Wu E, Yang H, Jiahui G, Duchaine B, Cook R (2017). Normal composite face effects in developmental prosopagnosia. Cortex.

[CR9] Blais C, Jack RE, Scheepers C, Fiset D, Caldara R (2008). Culture shapes how we look at faces. PLoS One.

[CR10] Bobak, A. K., Dowsett, A. J., & Bate, S. (2016a). Solving the border control problem: Evidence of enhanced face matching in individuals with extraordinary face recognition skills. *PLoS One*, *11*(2), e0148148. 10.1371/journal.pone.0148148 PMC4735453 26829321

[CR11] Bobak Anna K., Hancock Peter J. B., Bate Sarah (2015). Super-recognisers in Action: Evidence from Face-matching and Face Memory Tasks. Applied Cognitive Psychology.

[CR12] Bobak, A. K., Pampoulov, P., & Bate, S. (2016b). Detecting superior face recognition skills in a large sample of young British adults. *Frontiers in Psychology*, *7*, 1378. 10.3389/fpsyg.2016.01378. 10.3389/fpsyg.2016.01378 PMC5031595 27713706

[CR13] Bruce V, Bindemann M, Lander K (2018). Individual differences in face perception and person recognition. Cognitive Research: Principles and Implications.

[CR14] Bruce V, Henderson Z, Greenwood K, Hancock PJB, Burton AM, Miller P (1999). Verification of face identities from images captured on video. Journal of Experimental Psychology: Applied.

[CR15] Bruce V, Henderson Z, Newman C, Burton AM (2001). Matching identities of familiar and unfamiliar faces caught on CCTV Images. Journal of Experimental Psychology: Applied.

[CR16] Bruck, M., Cavanagh, P. (2018). Personal communication.

[CR17] Bruck M, Cavanagh P, Ceci SJ (1991). Fortysomething: Recognizing faces at one’s 25th reunion. Memory & Cognition.

[CR18] Burton AM, White D, McNeill A (2010). The Glasgow Face Matching Test. Behavior Research Methods.

[CR19] Burton AM, Wilson S, Cowan M, Bruce V (1999). Face recognition in poor-quality video: Evidence from security surveillance. Psychological Science.

[CR20] Busigny T, Joubert S, Felician O, Ceccaldi M, Rossion B (2010). Holistic perception of the individual face is specific and necessary: Evidence from an extensive case study of acquired prosopagnosia. Neuropsychologia.

[CR21] Duchaine B, Germine L, Nakayama K (2007). Family resemblance: Ten family members with prosopagnosia and within-class object agnosia. Cognitive Neuropsychology.

[CR22] Fysh MC (2018). Individual differences in the detection, matching and memory of faces. Cognitive Research: Principles and Implications.

[CR23] Fysh MC, Bindemann M (2018). The Kent Face Matching Test. British Journal of Psychology.

[CR24] Geskin J, Behrmann M (2018). Congenital prosopagnosia without object agnosia? A literature review. Cognitive Neuropsychology.

[CR25] Green W. (2019). Personal communication.

[CR26] Hancock PJB, Bruce V, Burton AM (2000). Recognition of unfamiliar faces. Trends in Cognitive Sciences.

[CR27] Heitz RP (2014). The speed-accuracy tradeoff: History, physiology, methodology, and behavior. Frontiers in Neuroscience.

[CR28] Herzmann G, Danthiir V, Schacht A, Sommer W, Wilhelm O (2008). Toward a comprehensive test battery for face cognition: Assessment of the tasks. Behavior Research Methods.

[CR29] Hildebrandt A, Herzmann G, Sommer W, Wilhelm O (2010). Structural invariance and age-related performance differences in face cognition. Psychology and Aging.

[CR30] Jenkins R, Burton AM (2011). Stable face representations. Philosophical Transactions of the Royal Society of London B: Biological Sciences.

[CR31] Jenkins R, White D, Van Montfort X, Burton AM (2011). Variability in photos of the same face. Cognition.

[CR32] Jenkins R., & Burton A. M. (2017). Personal communication.

[CR33] Lander K, Bruce V, Bindemann M (2018). Use-inspired basic research on individual differences in face identification: Implications for criminal investigation and security. Cognitive Research: Principles and Implications.

[CR34] Lao J, Vizioli L, Caldara R (2013). Culture modulates the temporal dynamics of global/local processing. Culture and Brain.

[CR35] Luce D (1986). Response times: Their role in inferring elementary mental organization.

[CR36] Marotta JJ, McKeeff TJ, Behrmann M (2002). The effects of rotation and inversion on face processing in prosopagnosia. Cognitive Neuropsychology.

[CR37] Megreya AM, Burton AM (2006). Unfamiliar faces are not faces: Evidence from a matching task. Memory & Cognition.

[CR38] Megreya AM, Sandford A, Burton AM (2013). Matching face images taken on the same day or months apart: The limitations of photo ID. Applied Cognitive Psychology.

[CR39] Moreton, R., Pike, G., & Havard, C. (2019). A task-and role-based perspective on super-recognizers: Commentary on ‘super-recognizers: From the laboratory to the world and back again’. *British Journal of Psychology* Early-Access. 10.1111/bjop.12394 30908603

[CR40] Noyes E, Hill MQ, O’Toole AJ (2018). Face recognition ability does not predict person identification performance: Using individual data in the interpretation of group results. Cognitive research: principles and implications.

[CR41] Noyes E, Phillips PJ, O'Toole AJ, Bindemann M, Megreya AM (2017). What is a super-recogniser?. Face processing: Systems, disorders, and cultural differences.

[CR42] Pachai, M. V., Sekuler, A. B., & Bennett, P. J. (2013). Sensitivity to information conveyed by horizontal contours is correlated with face identification accuracy. *Frontiers in Psychology*, *4*. 10.3389/fpsyg.2013.00074. 10.3389/fpsyg.2013.00074 PMC3580391 23444233

[CR43] Pachai MV, Sekuler AB, Bennett PJ, Schyns PG, Ramon M (2017). Personal familiarity enhances sensitivity to horizontal structure during processing of face identity. Journal of Vision.

[CR44] Papinutto M, Lao J, Ramon M, Caldara R, Miellet S (2017). The facespan—the perceptual span for face recognition. Journal of Vision.

[CR45] Phillips PJ, O'Toole AJ (2014). Comparison of human and computer performance across face recognition experiments. Image and Vision Computing.

[CR46] Phillips PJ, Yates AN, Beveridge JR, Givens G (2017). Predicting face recognition performance in unconstrained environments. 2017 IEEE Conference on Computer Vision and Pattern Recognition Workshops (CVPRW).

[CR47] Phillips PJ, Yates AN, Hu Y, Hahn CA, Noyes E, Jackson K (2018). Face recognition accuracy of forensic examiners, superrecognizers, and face recognition algorithms. Proceedings of the National Academy of Sciences.

[CR48] Puri KS, Suresh KR, Gogtay NJ, Thatte UM (2009). Declaration of Helsinki, 2008: Implications for stakeholders in research. Journal of Postgraduate Medicine.

[CR49] Ramon M (2018). The power of how—lessons learned from neuropsychology and face processing. Cognitive Neuropsychology.

[CR50] Ramon M (2018). Super-recognizers in law enforcement—hype or hope?. Invited symposium talk presented at the 29th International Congress of Applied Psychology (ICAP), Psychology: Connecting Science to Solutions, Montreal, Canada.

[CR51] Ramon Meike (2019). Super-Recognizers in Criminal Investigation – Hype or Hope?. Journal of Vision.

[CR52] Ramon Meike, Bobak Anna K., White David (2019). Super‐recognizers: From the lab to the world and back again. British Journal of Psychology.

[CR53] Ramon Meike, Bobak Anna K., White David (2019). Towards a ‘manifesto’ for super‐recognizer research. British Journal of Psychology.

[CR54] Ramon, M., Sokhn, N., & Caldara, R. (2019c). Decisional space modulates visual 15 categorization–Evidence from saccadic reaction times. Cognition, 186, 42-9. 10.1016/j.cognition.2019.01.019 30739058

[CR55] Ramon M, Busigny T, Rossion B (2010). Impaired holistic processing of unfamiliar individual faces in acquired prosopagnosia. Neuropsychologia.

[CR56] Ramon M, Gobbini MI (2018). Familiarity matters: A review on prioritized processing of personally familiar faces. Visual Cognition.

[CR57] Ramon M, Rossion B (2010). Impaired processing of relative distances between features and of the eye region in acquired prosopagnosia—two sides of the same holistic coin?. Cortex.

[CR58] Ramon M, Sokhn N, Caldara R (2019). Decisional space modulates visual categorization—evidence from saccadic reaction times. Cognition.

[CR59] Ramon Meike (2019). Super-Recognizers in Criminal Investigation – Hype or Hope?. Journal of Vision.

[CR60] Ramon, M., Sokhn, N., Lao, J., & Caldara, R. (2018). Decisional space determines saccadic reaction times in healthy observers and acquired prosopagnosia. Cognitive Neuropsychology, 35(5-6), 304-313. 10.1080/02643294.2018.1469482 29749293

[CR61] Ramon Meike (2019). Super-Recognizers in Criminal Investigation – Hype or Hope?. Journal of Vision.

[CR62] Ramon M, Van Belle G (2016). Real-life experience with personally familiar faces enhances discrimination based on global information. PeerJ.

[CR63] Rezlescu C, Pitcher D, Duchaine B (2012). Acquired prosopagnosia with spared within-class object recognition but impaired recognition of degraded basic-level objects. Cognitive Neuropsychology.

[CR64] Robertson, D. J., Noyes, E., Dowsett, A. J., Jenkins, R., & Burton, A. M. (2016). Face recognition by metropolitan police super-recognisers. PloSone, 11(2), e0150036. 10.1371/journal.pone.0150036 PMC4769018 26918457

[CR65] Robertson David James, Bindemann Markus (2019). Consolidation, wider reflection, and policy: Response to ‘Super‐recognisers: From the lab to the world and back again’. British Journal of Psychology.

[CR66] Russell R, Duchaine B, Nakayama K (2009). Super-recognizers: People with extraordinary face recognition ability. Psychonomic Bulletin & Review.

[CR67] Stacchi L, Liu-Shuang J, Ramon M, Caldara R (2019). Reliability of individual differences in neural face identity discrimination. NeuroImage.

[CR68] Sutherland CAM, Oldmeadow JA, Santos IM, Towler J, Michael Burt D, Young AW (2013). Social inferences from faces: Ambient images generate a three-dimensional model. Cognition.

[CR69] Tarr MJ, Peterson MA, Rhodes G (2003). Visual object recognition: Can a single mechanism suffice?. Advances in visual cognition. Perception of faces, objects, and scenes: Analytic and holistic processes.

[CR70] Townsend JT, Ashby FG (1978). Methods of modeling capacity in simple processing systems. Cognitive Theory.

[CR71] Tüttenberg Simone C, Wiese Holger (2019). Learning own- and other-race facial identities from natural variability. Quarterly Journal of Experimental Psychology.

[CR72] Watier NN, Collin CA (2009). Effects of familiarity on spatial frequency thresholds for face matching. Perception.

[CR73] White D, Kemp RI, Jenkins R, Matheson M, Burton AM (2014). Passport officers’ errors in face matching. PLoS One.

[CR74] White D, Phillips PJ, Hahn CA, Hill M, O’Toole AJ (2015). Perceptual expertise in forensic facial image comparison. Proceedings of the Royal Society B: Biological Sciences.

[CR75] White D, Rivolta D, Mike Burton A, Al-Janabi S, Palermo R (2017). Face matching impairment in developmental prosopagnosia. Quarterly Journal of Experimental Psychology.

[CR76] Young AW, Burton AM (2017). Recognizing faces. Current Directions in Psychological Science.

