# Supplementary figures and images for: Normative data for two challenging tests of face matching under ecological conditions
Source: Cogn Res Princ Implic. 2020 Feb 19;5:8. doi: 10.1186/s41235-019-0205-0 (PMC7031457; doi:10.1186/s41235-019-0205-0)

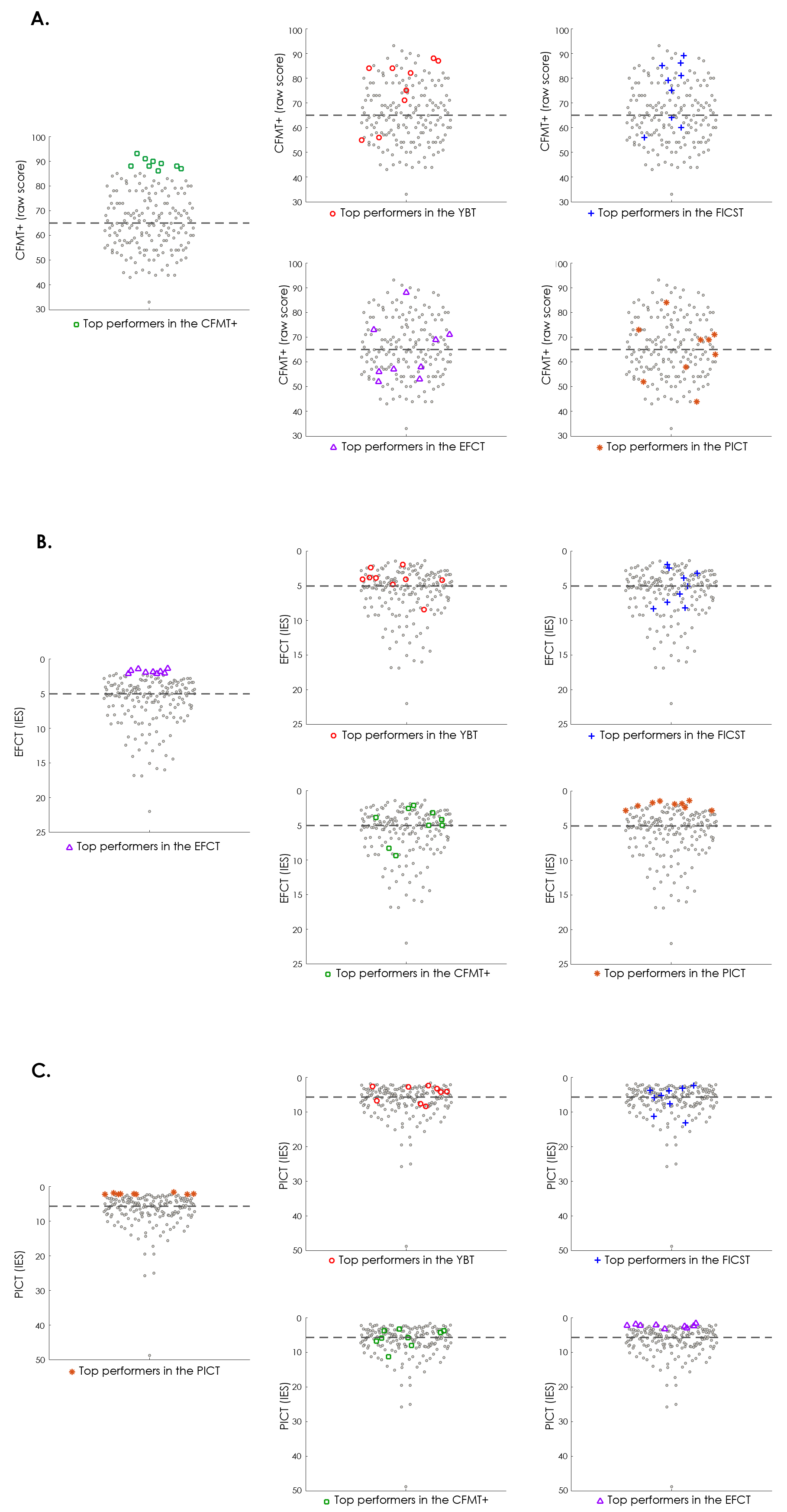

Supplement: Supplementary file 1 — Additional file 1: Figure S1. CFMT+, EFCT and PICT scores of the top performers identified independently per test. Visualized here are performance levels of individuals identified as the top performers (colored markers) based on the YBT (red circles), FICST (blue crosses), CFMT+ (green squares), EFCT (purple triangles), and PICT (orange asterisks), and their relative location among all observations made (grey dots) for the a CFMT+, b EFCT and c PICT. [file 41235_2019_205_MOESM1_ESM.tif]
